# On Modern Progress in Ophthalmic Medicine and Surgery

**Published:** 1894-04-07

**Authors:** Robert Brudenell Carter

**Affiliations:** Consulting Ophthalmic Surgeon to St. George's Hospital


### On Modern Progress in Ophthalmic Medicine and Surgery.

By Robert Brudenell Carter, F.R.C.S., Consulting Ophthalmic Surgeon to St. George's Hospital.

Von Graefe, having thus classified the causes of suppuration after cataract extraction, proceeded to consider by what means these causes might be set aside. Regarding the iritis as an effect of mechanical injury, it naturally occurred to him to suggest that it would be less likely to occur in an eye upon which iridectomy had been previously performed, so that the lens might pass easily through the resulting gap in the iris tissue. His chief attention, however, seems to have been given to the cornea. This structure, in old or emaciated people, often becomes very thin and feeble; insomuch that, when deprived of support from the lens and aqueous humour, it falls easily into folds or wrinkles, and does not retain sufficient stiffness to insure accurate contact of the edges of the flap-operation wound, the duehealing of which then becomes impossible. Moreover, having no blood vessels of its own, and hence deriving the materials of its nutrition from adjacent tissues, it is liable to slough merely from the fact of having one half of its connections with these tissues interrupted by an incision. In view of these conditions, Von Graefe applied his mind chiefly to the possibility of so placing the external wound that it should lie in vascular tissue, and that it should not be disposed to gape spontaneously.

While he was occupied by endeavours to solve this problem, one of his pupils, Dr. Mooren, began to carry out the suggestion of a preliminary iridectomy, which he performed at first a fortnight, and afterwards six weeks, prior to the accomplishment of extraction in the ordinary way. The results of this procedure were so successful that Mooren published them in a pamphlet. He obtained prompt healing and good vision in a number of old and emaciated peasants, whose cornea became wrinkled when the anterior chamber was opened, and whose eyes would almost inevitably have perished but for the preliminary iridectomy. His pamphlet was received in Germany with so much incredulity that he issued a second edition, in which he gave the name and address of every patient upon whom he had operated in the manner described, and he invited his readers to go and examine these people for themselves. The adoption of such a course seemed to throw a curious light upon the German standard of professional integrity; for it is hardly possible to imagine that anything analogous could ever be done by a self-respecting Englishman. Mooren sent me his pamphlet, and I believe I was (in 1862) the first surgeon in England to practise his operation. I selected for it the second eyes of patients upon whom I had previously operated successfully by the flap method, and I was thoroughly satisfied with the results, both surgical and visual. The proceeding was, however, attended by the manifest disadvantage of requiring two operations and two periods of confinement in order to do what had previously been done in a single one. Still, it was undeniable that the preliminary iridectomy prevented bruising of the iris or stretching of the pupil, and also that it permitted the exit of the lens through a smaller external section, thus diminishing, in a corresponding degree, the risk of sloughing of the cornea.

While Mooren was proceeding in this manner, Von Graefe was endeavouring to bring to perfection his own new method of operating, which he ultimately described as "modified linear extraction." In this the incision was made wholly outside the true cornea, in the zone of tissue immediately in front of the iris, and in a plane passing from the corneal margin to the centre of the eyeball. Hence its centre was somewhat anterior to its extremities, and its position was such that its edges fell naturally into perfect apposition. An iridectomy, instead of being performed beforehand, was made the second step of the operation. The small available space rendered the external incision short as compared with that of flap extraction, and it was soon found that the pressure necessary for the extrusion of the lens through a small opening was liable to be attended by loss of vitreous, which, although in consequence of the iridectomy it no longer lifted the iris into the external wound, was still a serious and undesirable complication. Hence Von Graefe introduced a curved hook, which he passed into the eye, carried under the lower margin of the lens, and then employed as a traction instrument so as to pull out the cataract without having recourse to pressure. He was followed, after a time, by Dr. Schuft, who replaced the hook by a spoon of oval outline, with a margin all round at right angles to its floor. Schuft described his method as the "out-scooping" (Auslöffelung) of cataract, and followed the example of Mooren by publishing a pamphlet. His spoon found a brief acceptance at Moorfields, where the late Sir William Bowman and the late Mr. Critchett were engaged in earnest endeavours to separate any grain yielded by the German experiments from the chaff. At Moorfields the spoon was soon modified by the loss of all but the front portion of its margin, Sir William inclining this front portion forwards to permit it to pass more easily under the lens; while Mr. Critchett inclined it backwards, like the toe of a slipper, to give a better hold for the purpose of extraction. Before long the results of the use of traction instruments in any form were found to be so disastrous that they were abandoned; and the practical outcome of several years of experimentation was to replace the old incision by one lying just outside of the cornea, with its centre a little anterior to its extremities, to make this incision long enough to permit of the easy exit of the lens, and to perform iridectomy as part of the operation. It is a curious feature of the history that it affords what is, perhaps, the only example of the employment of vivisection on a large scale upon the human subject. Von Graefe was able to establish the efficacy of iridectomy, as a means of reducing ocular tension, by experimental operations upon the lower animals; but, on account of anatomical differences, it was impossible to use them for the purpose of determining the relative advantages of different methods of extracting cataract from the human subject. The consequence was that the experiments necessary for the latter purpose were performed upon men and women, that the men and women so employed were not, as a rule, informed that they were to be operated upon by an experimental method, and that the destruction of eyes, especially in Germany, during the period over which the experimentation continued, was probably such as to reduce Beer's traditional "hatful" to utter insignificance by comparison.

